# Cerebral Venous Sinus Thrombosis (CVST): A Clinically Significant Neurological Condition

**DOI:** 10.7759/cureus.62700

**Published:** 2024-06-19

**Authors:** Utkarsh Gaur, Charuta Gadkari, Aditya Pundkar

**Affiliations:** 1 Emergency Medicine, Jawaharlal Nehru Medical College, Datta Meghe Institute of Higher Education and Research, Wardha, IND; 2 Orthopedics, Jawaharlal Nehru Medical College, Datta Meghe Institute of Higher Education and Research, Wardha, IND

**Keywords:** case report, headache, patient, sinus, cavernous venous sinus thrombosis

## Abstract

Cerebral venous sinus thrombosis (CVST), a rare but deadly disorder, causes papilledema as well as a number of frequent clinical symptoms, including excruciating headaches, focal seizures, and paralysis on one or both sides of the body.

In this intriguing case study, we present the clinical narrative of a 45-year-old man who sought medical attention due to severe headaches persisting for two days. Concurrently, he experienced an abrupt onset of tingling and numbness in his left upper arm. Remarkably, magnetic resonance venography (MRV) revealed an absence of the sigmoid sinus, left transverse sinus, left jugular vein, and superior sagittal sinus, adding complexity to the diagnostic puzzle. Despite this anomaly, conventional brain MRI findings appeared normal. The patient reported a significant reduction in headache intensity following treatment, which included a year-long course of anticoagulant therapy. Subsequently, he gradually regained his health, underscoring the importance of multidisciplinary approaches in managing such challenging cases.

This example emphasizes the significance of considering CVST while developing a differential diagnosis of various neurological disorders. Given the vast spectrum of clinical symptoms associated with CVST, it should be taken into account as a potential causative factor in a number of neurological illnesses, in order for patients to experience the best outcomes, quick diagnosis, and quality care.

## Introduction

Less than 1% of all stroke cases are caused by cerebral venous sinus thrombosis (CVST), a rare but serious neurological condition that primarily affects young people and children [1-4]. This condition can present with a variety of clinical signs and symptoms, including severe headaches, nausea, vomiting, ocular papilledema, limb weakness, and seizures. Despite the severity of CVST, misdiagnosis rates have been estimated to reach 50% [5]. The intricate and varied clinical manifestations of CVST, coupled with its rarity, contribute to the diagnostic complexities associated with this ailment [6].

Clinical presentation and challenges

While headache is the most prevalent symptom among the diverse clinical presentations of CVST, rapidly progressive dementia (RPD) is surprisingly rare [7,8]. The case report presents a patient who was well-oriented in time, place, and person, with no clinical findings suggestive of cognitive decline or decreased responsiveness. Ultimately, this led to the diagnosis of CVST. This can be attributed to the unusual presentation and relative lack of awareness surrounding CVST as a potential differential diagnosis.

Objective of case report

This case report endeavors to elucidate the patient’s clinical presentation, examination findings, diagnostic procedures, and treatment, emphasizing the importance of considering unusual neurological conditions, even when patients exhibit atypical risk factors.

## Case presentation

A 45-year-old man who had experienced terrible headaches for the previous two days, as well as abrupt tingling and numbness in his left upper limb, went to the emergency room. Upon assessment, his vital signs were steady, including his blood pressure of 160/90 mmHg, oxygen saturation of 99% (SpO2), and respiratory rate of 16 breaths per minute. With a Glasgow Coma Scale (GCS) of 15, he was awake and alert. He had a random blood sugar level of 112 mg/dL and showed no signs of discomfort or fever.

The patient complained of a sudden, severe headache that would not go away with painkillers. Along with the headache and nausea, he also noticed weakness and tingling in his left upper limb the day he was admitted. It was significant that the weakness did not get worse and that he still had enough strength to lift his arm against gravity. He had no prior history of seizures, mental instability, dysphagia, vertigo, tinnitus, diplopia, slurred speech, or loss of consciousness.

The patient’s last alcohol consumption was four days before admission, and he had a lengthy 15-year history of chronic alcoholism, diabetes, hypertension, tuberculosis, asthma, or chronic obstructive pulmonary disease that were not present in the past.

The patient had a GCS score of 15 during the neurological test, with both pupils measuring 3 mm and responding to light. In the motor assessment, the left upper limb’s strength was reduced (3/5), whereas the right upper limb and both lower limbs had normal strength (5/5). Ankle, knee, biceps, and triceps deep tendon reflexes were brisk, and the plantar reflexes were bilaterally flexor. A sensory evaluation found no deficiencies in the perception of pain or warmth. The finger-nose test, heel-shin test, dysdiadochokinesia, and assessment of the cranial nerves were all clear.

Laboratory tests showed a hemoglobin level of 12.3 g/dL, a normal coagulation profile, and slight anemia. Liver function tests revealed serum glutamic oxaloacetic transaminase (SGOT) and serum glutamic pyruvic transaminase (SGPT) levels of 52 U/L and 45 U/L, respectively, with a total protein of 6.3 g/dL and albumin of 3.5 g/dL. Kidney function tests fell within normal ranges. D-dimer levels were 754 ng/mL.

**Figure 1:**
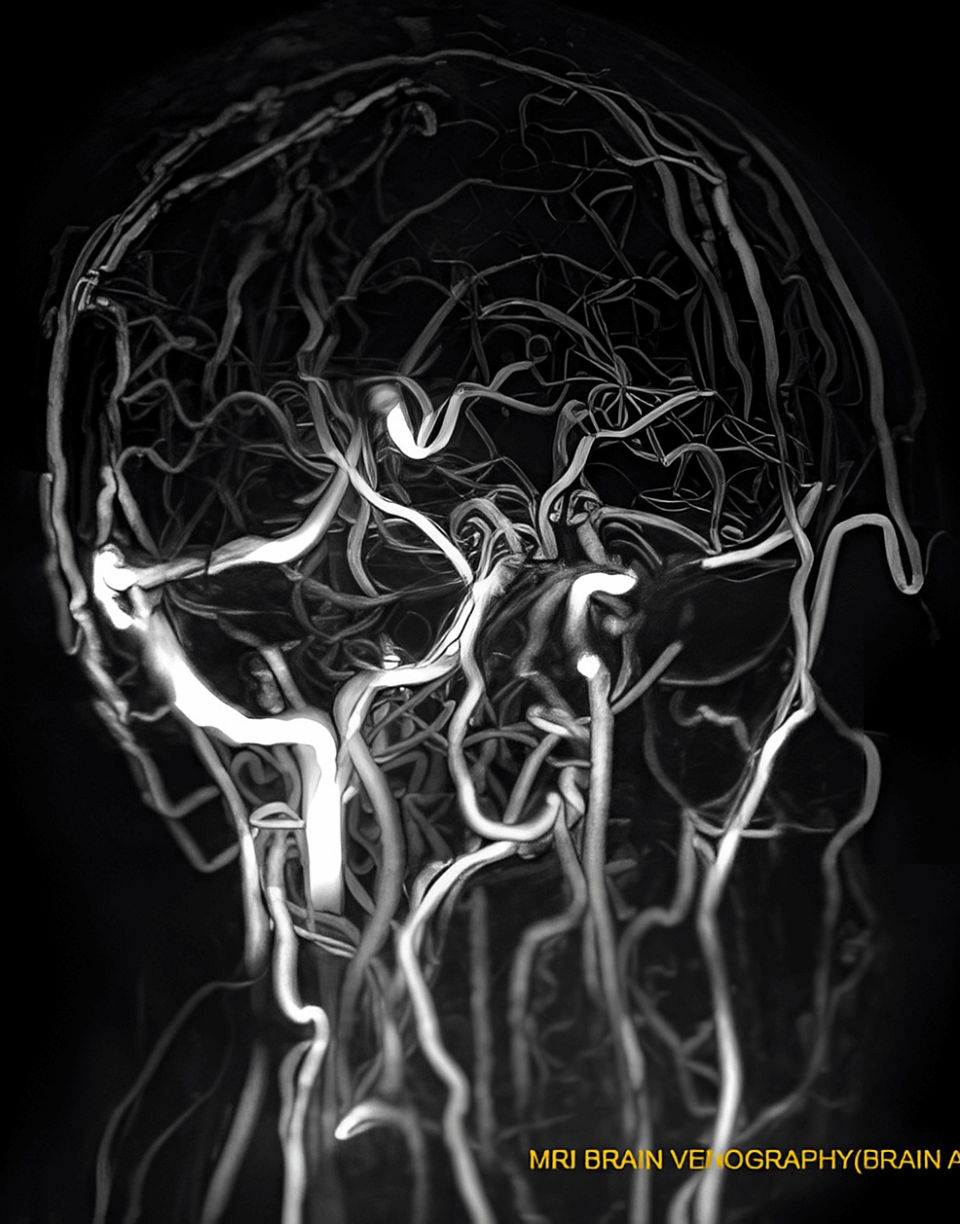
Magnetic resonance venogram (MRV) showing non-visualization of the sinuses due to thrombosis within them This image indicates non-visualization of the superior sagittal sinus, inferior sagittal sinus, left transverse sinus, sigmoid sinus, and left jugular vein due to thrombosis. This observation suggests the presence of cerebral venous sinus thrombosis.

**Figure 2:**
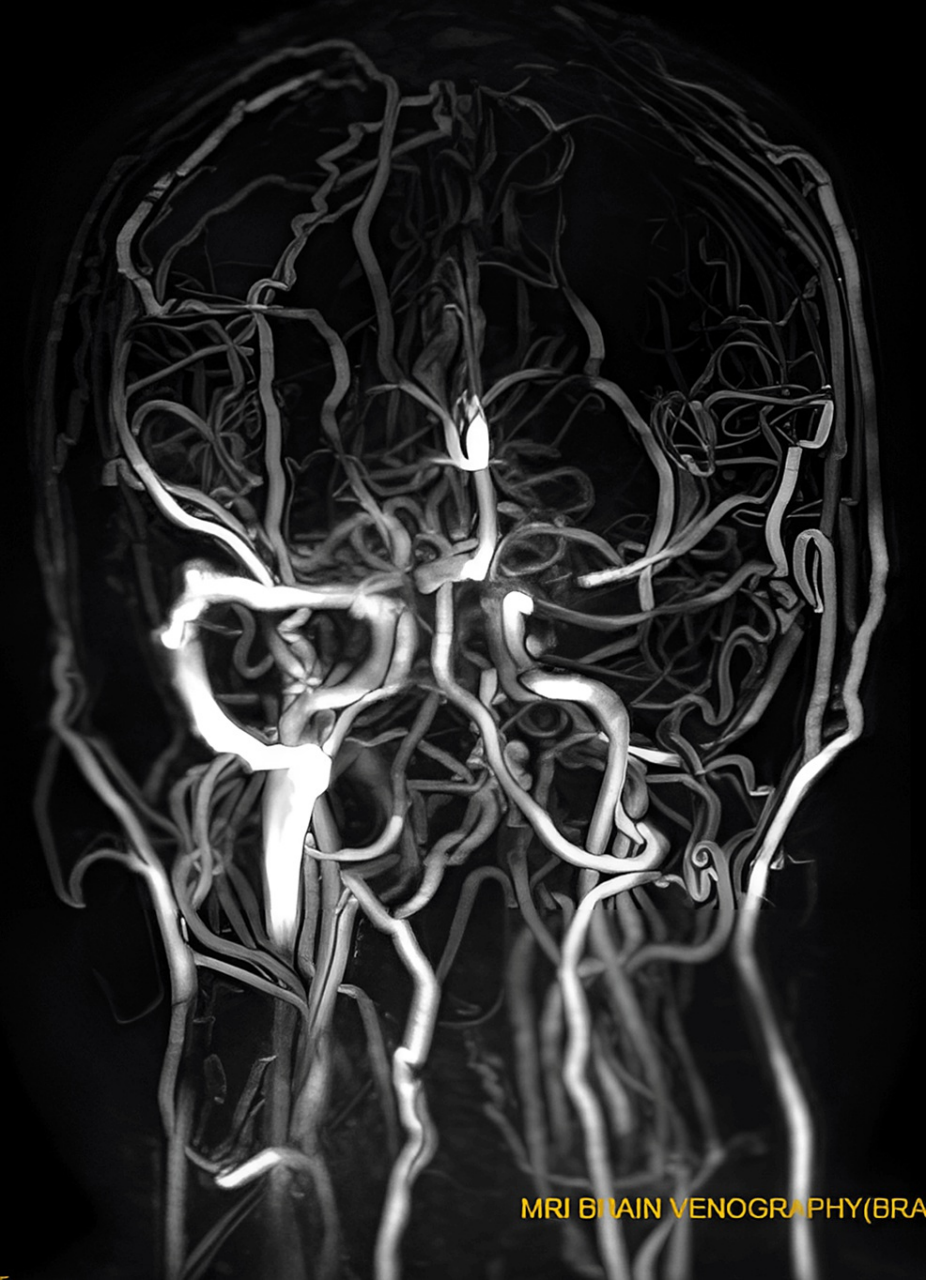
Another magnetic resonance venogram (MRV) image showing loss of flow signal in the major venous sinuses This image demonstrates the absence of flow signal in the superior sagittal sinus, inferior sagittal sinus, left transverse sinus, sigmoid sinus, and left jugular vein, implying thrombosis within these sinuses and indicating the presence of cerebral venous sinus thrombosis.

Magnetic resonance venography (MRV) images did not show important venous sinuses, including the superior sagittal sinus, inferior sagittal sinus, left transverse sinus, sigmoid sinus, and left jugular vein (see Figures 1, 2). However, brain MRI results were normal.

Based on the clinical picture and the MRV test results, the patient was diagnosed with CVST. As urgent therapies, subcutaneous low molecular weight heparin (LMWH) 60 mg, intravenous thiamine 500 mg, iv paracetamol 1 g, and iv levetiracetam 1 g were employed. Iv pantoprazole 40 mg and iv ondansetron 4 mg were provided with supportive care. The patient was transferred to the neurology critical care unit (ICU) for close monitoring, to continue taking anticoagulants and antiepileptic drugs, as well as for additional supportive therapies.

During the one-week stay in the neurology ICU, the patient experienced a notable reduction in headache severity. He was oriented to time, place, and person and was consistent with ICU medications. Remarkably, the patient’s muscle strength improved to 4/5, accompanied by diminished tingling sensations in the left upper limb. Upon discharge, the patient was prescribed a regimen comprising tab. levetiracetam 500 mg BD, tab. amitriptyline hydrochloride 10 mg before bed, tab. dabigatran 150 mg BD, tab. paracetamol 650 mg SOS, and tab. supradyn OD. A subsequent follow-up after two weeks revealed sustained orientation, absence of tingling sensations in the left upper limb, commendable compliance with the prescribed medications, and the continued alleviation of headache symptoms. Additionally, the patient was advised to undergo one week of physiotherapy to further enhance recovery.

## Discussion

CVST, an uncommon but clinically significant neurological condition, primarily affects adolescents and children. Although a severe headache is the hallmark sign of CVST, this illness can also present with seizures, altered awareness, focal neurological impairments, vertigo, and other clinical manifestations [8]. Although headaches are typically the first sign of CVST, this case report stresses the rarity and quick progression of dementia as the condition’s first symptom [9].

RPD is characterized by a swift decline in cognitive function that progresses rapidly to dementia, often presenting opportunities for partial reversibility and treatment [10]. RPD manifests in two main forms: primary and secondary. Primary RPD typically stems from rapidly progressive neurodegenerative disorders, notably Creutzfeldt-Jakob disease (CJD). Conversely, secondary RPD, linked to swiftly advancing central nervous system (CNS) conditions, can result from various pathologies, such as Lewy body disease or cerebrovascular issues, alongside Alzheimer’s disease. The pace of progression and potential for reversibility in secondary RPD vary due to multifactorial influences [11]. In this particular instance, the patient did not exhibit any deterioration in cognitive function that matched the symptoms of CJD, and several other diagnostic requirements were not satisfied.

Idiopathic intracranial hypertension (IIH) and secondary intracranial hypertension are two types of elevated intracranial pressure. IIH frequently causes symptoms like headaches, optic papilledema, and visual abnormalities in obese women of reproductive age [12]. In contrast, secondary intracranial hypertension has a known cause, such as lesions that take up space or thrombotic conditions [13,14]. The patient had elevated cerebrospinal fluid (CSF) pressure, but there were noticeably no characteristic IIH symptoms. Therefore, neither high intracranial pressure associated with other recognized etiologies nor IIH was able to fully explain the patient’s situation [15].

Due to its varied clinical manifestations, CVST is still identified as a challenging conundrum. CVST cannot be completely ruled out, not even in situations where cranial MRI and head CT appear to be normal. Additional MRV screening is frequently necessary as a result [16,17]. CVST has been diagnosed and prognosed with outstanding accuracy by combining cranial MRI and MRV [18].

The pathogenesis of CVST depends on a complex interplay between venous thrombosis and fibrinolytic processes. CVST is caused by a variety of infectious and non-infectious mechanisms, including hereditary prothrombotic illnesses, autoimmune diseases, cancers, and other conditions [19]. An increasing body of research has also shown a connection between COVID-19 infection and CVST [20-23]. While not conclusive in this case, the patient’s elevated D-dimer levels (754 ng/mL) were consistent with CVST, highlighting the potential value of D-dimer evaluation in the diagnosis of CVST [24,25].

The complexity of diagnosing CVST, a disorder that might present with unusual symptoms such as a strong headache and neurological impairments, is highlighted by this case report. Although a severe headache is the most typical sign, medical professionals must be aware that CVST can present itself in a variety of ways. The risk of CVST is not eliminated just because the typical headache is absent. Numerous neurological symptoms, such as vertigo, altered mental status, focal impairments, and seizures, may be present in patients. Therefore, while assessing people with unexplained neurological problems, doctors should keep a high index of suspicion and use a broad diagnostic range.

The importance of a thorough evaluation is shown by this example, especially in individuals with underlying problems like chronic alcoholism. The fact that chronic drinking can be a predisposing factor further emphasizes the importance of thorough assessment and early management in such situations. Chronic drinking may contribute to a hypercoagulable condition and other physiological changes that enhance the likelihood of thrombotic events, even if it is not a direct risk factor for CVST [26]. When assessing patients who have ambiguous neurological symptoms, these risk factors must be considered because they can offer helpful diagnostic hints.

CVST must be adequately managed, and this requires early diagnosis and fast treatment. The prognosis can get worse, and problems can arise from delayed recognition. As a result, medical personnel should be on the lookout for unusual neurological symptom presentations and not ignore them. A thorough assessment that incorporates cutting-edge imaging, such as cranial MRI and MRV, can help arrive at a prompt and precise diagnosis.

The cornerstone of medical practice is ensuring the best possible patient care. CVST can take many different forms, and it is important to identify it and treat it right away for the best possible results and patient welfare. By identifying the possibility of CVST in atypical headache presentations, healthcare professionals uphold their dedication to providing thorough and patient-centered care.

The urgent need for early diagnosis of CVST and adequate management emphasizes the need for medical personnel to be aware of and vigilant about this uncommon but potentially severe neurological condition.

## Conclusions

Even in the absence of conventional risk factors, CVST should be taken into account in patients who arrive with atypical headache symptoms. In order to enhance the prognosis and guarantee of the best possible patient care, this example highlights the significance of a complete evaluation and early intervention, especially for people with underlying problems like persistent alcoholism.
